# Author Correction: Cellular deconvolution of GTEx tissues powers discovery of disease and cell-type associated regulatory variants

**DOI:** 10.1038/s41467-020-18341-8

**Published:** 2020-09-01

**Authors:** Margaret K. R. Donovan, Agnieszka D’Antonio-Chronowska, Matteo D’Antonio, Kelly A. Frazer

**Affiliations:** 1 Bioinformatics and Systems Biology Graduate Program, University of California, San Diego, La Jolla, CA 92093 USA; 2 Department of Biomedical Informatics, University of California, San Diego, La Jolla, CA 92093 USA; 3 Department of Pediatrics and Rady Children’s Hospital, University of California, San Diego, La Jolla, CA 92093 USA; 4 Institute for Genomic Medicine, University of California, San Diego, La Jolla, CA 92093 USA

**Keywords:** Gene expression, Genetic association study, RNA sequencing

Correction to: *Nature Communications* 10.1038/s41467-020-14561-0, published online 19 February 2020.

The original version of the Supplementary Information associated with this Article included incomplete Supplementary Data files 19, 20, 21, 23, 24, which did not include the full cell-type composition statistics. The HTML has been updated to include expanded versions of Supplementary Data 19, 20, 21, 23, 24; the original versions of Supplementary Data 19, 20, 21, 23, 24 can be found as Supplementary Information associated with this Correction.

## Supplementary information

Supplementary Data 19

Supplementary Data 20

Supplementary Data 21

Supplementary Data 23

Supplementary Data 24

