# Supplementary material for: Cellular deconvolution of GTEx tissues powers discovery of disease and cell-type associated regulatory variants
Source: Nat Commun. 2020 Feb 19;11:955. doi: 10.1038/s41467-020-14561-0 (PMC7031340; doi:10.1038/s41467-020-14561-0)
Supplement: Supplementary file 1 — Supplementary Information [file 41467_2020_14561_MOESM1_ESM.docx]

# Supplementary Data file legends

## Supplementary Data 1 legend. Signature genes from cell populations identified in 14 scRNA-seq tissues

For each scRNA-seq dataset (14 organs from mouse and two tissues from human) used to deconvolute GTEx tissues, we extracted signature genes. In Supplementary Data 1A, for each organ/tissue, (Column A), species (Column B), cell type (Column C), the signature gene ensemble IDs (signature_gene_ids; Column D) are given. In subsequent tables (Supplementary Data 1B-Q), the mean signature gene expressions from each cell type comprising each organ/tissue/species dataset are given.

## Supplementary Data 2 legend. Correlation of cell type estimates obtained from human versus mouse expression signatures

The table shows, for each combination of cell type from human (column A) and mouse (column B), the observed correlation (column C), the mean and standard deviation of correlations in 1,000 permutations (column D, E), the Z-score (calculated as the difference between observed correlation and mean correlation in the permutations, divided by the standard deviation, column F), the empirical p-value (calculated using as the number of permutations with correlation greater than the observed value, divided by the number of permutations + 1, column G) and the Benjamini-Hochberg-adjusted p-value (column H). Panel A shows liver cell types, while panel B shows skin cell types.

## Supplementary Data 3 legend. Simulation analysis to examine accuracy of deconvolution using human and mouse signature genes

To examine the accuracy of the deconvolution using both human and mouse signature genes, we used human liver scRNA-seq data to obtain 100 simulated samples with known cell type distributions. The table shows, for each simulated sample: the number of cells from each cell type used to obtain the mixture (column B-P); 2) the cell type estimates using human expression signatures (column Q-AE); 3) cell type estimates using mouse expression signatures (column AF-AJ); 4) known collapsed cell type distributions (column AK-AQ); and 5) cell type estimates using human collapsed expression signatures (column AR-AX). For the known cell type distributions the number of cells is shown, while for the cell type estimates the relative distributions are displayed.

## Supplementary Data 4 legend. Cellular composition estimates of bulk RNA-seq from GTEx Artery (Aorta) using mouse aorta signature genes

## Supplementary Data 5 legend. Cellular composition estimates of bulk RNA-seq from GTEx Heart (Atrial appendage) using mouse heart (subsetted to atrium cells) signature genes

Input Sample (Column A) indicates the deconvoluted GTEx RNA-seq sample. Subsequent columns indicate the fraction of each estimated cell populations, and CIBERSORT p-value.

## Supplementary Data 6 legend. Cellular composition estimates of bulk RNA-seq from GTEx Brain (Amygdala, Anterior cingulate cortex (BA24), Caudate (basal ganglia), Cerebellar Hemisphere, Cerebellum, Cortex, Frontal Cortex (BA9), Hippocampus, Hypothalamus, Nucleus accumbens (basal ganglia), Putamen (basal ganglia), Spinal cord (cervical c-1), Substantia nigra) using mouse brain non-microglia signature genes

Input Sample (Column A) indicates the deconvoluted GTEx RNA-seq sample. Subsequent columns indicate the fraction of each estimated cell populations, and CIBERSORT p-value.

## Supplementary Data 7 legend. Cellular composition estimates of bulk RNA-seq from GTEx Colon (Sigmoid and transverse) using mouse colon signature genes

Input Sample (Column A) indicates the deconvoluted GTEx RNA-seq sample. Subsequent columns indicate the fraction of each estimated cell populations, and CIBERSORT p-value.

## Supplementary Data 8 legend. Cellular composition estimates of bulk RNA-seq from GTEx Adipose (Subcutaneous, Visceral (Omentum)) using mouse fat signature genes

Input Sample (Column A) indicates the deconvoluted GTEx RNA-seq sample. Subsequent columns indicate the fraction of each estimated cell populations, and CIBERSORT p-value.

## Supplementary Data 9 legend. Cellular composition estimates of bulk RNA-seq from GTEx Liver using human liver signature genes

Input Sample (Column A) indicates the deconvoluted GTEx RNA-seq sample. Subsequent columns indicate the fraction of each estimated cell populations, and CIBERSORT p-value.

## Supplementary Data 10 legend. Cellular composition estimates of bulk RNA-seq from GTEx Liver using mouse liver signature genes

Input Sample (Column A) indicates the deconvoluted GTEx RNA-seq sample. Subsequent columns indicate the fraction of each estimated cell populations, and CIBERSORT p-value.

## Supplementary Data 11 legend. Cellular composition estimates of bulk RNA-seq from GTEx Breast (mammary) using mouse mammary signature genes

Input Sample (Column A) indicates the deconvoluted GTEx RNA-seq sample. Subsequent columns indicate the fraction of each estimated cell populations, and CIBERSORT p-value.

## Supplementary Data 12 legend. Cellular composition estimates of bulk RNA-seq from GTEx Muscle (skeletal muscle) using mouse muscle signature genes

Input Sample (Column A) indicates the deconvoluted GTEx RNA-seq sample. Subsequent columns indicate the fraction of each estimated cell populations, and CIBERSORT p-value.

## Supplementary Data 13 legend. Cellular composition estimates of bulk RNA-seq from GTEx Pancreas using mouse pancreas signature genes

Input Sample (Column A) indicates the deconvoluted GTEx RNA-seq sample. Subsequent columns indicate the fraction of each estimated cell populations, and CIBERSORT p-value.

## Supplementary Data 14 legend. Cellular composition estimates of bulk RNA-seq from GTEx Skin (Not sun exposed (Suprapubic), Sun exposed (Lower leg)) using mouse skin signature genes

Input Sample (Column A) indicates the deconvoluted GTEx RNA-seq sample. Subsequent columns indicate the fraction of each estimated cell populations, and CIBERSORT p-value.

## Supplementary Data 15 legend. Cellular composition estimates of bulk RNA-seq from GTEx Skin (Not sun exposed (Suprapubic), Sun exposed (Lower leg)) using human epidermis signature genes

Input Sample (Column A) indicates the deconvoluted GTEx RNA-seq sample. Subsequent columns indicate the fraction of each estimated cell populations, and CIBERSORT p-value.

## Supplementary Data 16 legend. Cellular composition estimates of bulk RNA-seq from GTEx Spleen using mouse spleen signature genes

Input Sample (Column A) indicates the deconvoluted GTEx RNA-seq sample. Subsequent columns indicate the fraction of each estimated cell populations, and CIBERSORT p-value.

## Supplementary Data 17 legend. Cellular composition estimates of bulk RNA-seq from GTEx Heart (Left ventricle) using mouse heart (subsetted to ventricle cells) signature genes

Input Sample (Column A) indicates the deconvoluted GTEx RNA-seq sample. Subsequent columns indicate the fraction of each estimated cell populations, and CIBERSORT p-value.

## Supplementary Data 18 legend. eQTL results: GTEx liver at bulk resolution

The ensemble gene ID (Column A), the gene name (Column B), the start (Column C) and end (Column D) of the gene, the gene ID (chromosome_start_end_genotype; Column E), variant position (Column F), reference allele (Column G), alternative allele (Column H), the reference SNP cluster ID (Column I), delta AIC (Column J) and p-value (Column K) for covariates included in the model, and FDR (Column L) are included.

## Supplementary Data 19 legend. eQTL results: GTEx liver at high resolution

The ensemble gene ID (Column A), the gene name (Column B), the start (Column C) and end (Column D) of the gene, the gene ID (chromosome_start_end_genotype; Column E), variant position (Column F), reference allele (Column G), alternative allele (Column H), the reference SNP cluster ID (Column I), delta AIC (Column J) and p-value (Column K) calculated using endothelial venous cell composition, the delta AIC (Column L) and p-value (Column M) calculated using delta cell composition, the delta AIC (Column N) and p-value (Column O) calculated using hepatocyte0 cell composition, the delta AIC (Column P) and p-value (Column Q) calculated using hepatocyte3 cell composition, the delta AIC (Column R) and p-value (Column S) calculated using hepatocyte4 cell composition, the delta AIC (Column T) and p-value (Column U) calculated using macrophage cell composition, the delta AIC (Column V) and p-value (Column W) calculated using NK/T cell composition, the delta AIC (Column X) and p-value (Column Y) calculated using endothelial periportal cell composition, the q-values calculated using each cell composition (Column Z-AG), and the number of cell types with significant associations. All the eGenes with only one significant cell type association were considered as “cell type-specific”, whereas all the eGenes with more than one significant cell type association were considered as “cell type-associated”..

## Supplementary Data 20 legend. eQTL results: GTEx liver at collapsed resolution

The ensemble gene ID (Column A), the gene name (Column B), the start (Column C) and end (Column D) of the gene, the gene ID (chromosome_start_end_genotype; Column E), variant position (Column F), reference allele (Column G), alternative allele (Column H), the reference SNP cluster ID (Column I), delta AIC (Column J) and p-value (Column K) calculated using merged hepatocytes cell composition, the delta AIC (Column L) and p-value (Column M) calculated using merged endothelial cells composition, the delta AIC (Column N) and p-value (Column O) calculated using merged macrophage cell composition, the delta AIC (Column P) and p-value (Column Q) calculated using merged B cells composition, the delta AIC (Column R) and p-value (Column S) calculated using merged NK and NK/T cells composition, the delta AIC (Column T) and p-value (Column U) calculated using merged cholangiocytes cell composition, the delta AIC (Column V) and p-value (Column W) calculated using merged hepatic stellate cell composition, the q-values calculated using each cell composition (Columns X-AC), and the number of cell types with significant associations (Column AD). All the eGenes with only one significant cell type association were considered as “cell type-specific”, whereas all the eGenes with more than one significant cell type association were considered as “cell type-associated”.

## Supplementary Data 21 legend. eQTL results: GTEx liver at low resolution

The ensemble gene ID (Column A), the gene name (Column B), the start (Column C) and end (Column D) of the gene, the gene ID (chromosome_start_end_genotype; Column E), variant position (Column F), reference allele (Column G), alternative allele (Column H), the reference SNP cluster ID (Column I), delta AIC (Column J) and p-value (Column K) calculated using endothelial cell composition, the delta AIC (Column L) and p-value (Column M) calculated using hepatocyte cell composition, the delta AIC (Column N) and p-value (Column O) calculated using Kupffer cell composition, the delta AIC (Column P) and p-value (Column Q) calculated using NK cell composition, the q-values calculated using each cell composition (Columns R-U), and the number of cell types with significant associations (Column V). All the eGenes with only one significant cell type association were considered as “cell type-specific”, whereas all the eGenes with more than one significant cell type association were considered as “cell type-associated”.

## Supplementary Data 22 legend. eQTL results: GTEx skin at bulk resolution

The ensemble gene ID (Column A), the gene name (Column B), the start (Column C) and end (Column D) of the gene, the gene ID (chromosome_start_end_genotype; Column E), variant position (Column F), reference allele (Column G), alternative allele (Column H), the reference SNP cluster ID (Column I), delta AIC (Column J) and p-value (Column K) for covariates included in the model, and FDR (Column L) are included.

## Supplementary Data 23 legend. eQTL results: GTEx skin at high resolution

The ensemble gene ID (Column A), the gene name (Column B), the start (Column C) and end (Column D) of the gene, the gene ID (chromosome_start_end_genotype; Column E), variant position (Column F), reference allele (Column G), alternative allele (Column H), the reference SNP cluster ID (Column I), delta AIC (Column J) and p-value (Column K) calculated using epidermis cell composition, the delta AIC (Column L) and p-value (Column M) calculated using outer bulge cell composition, the delta AIC (Column N) and p-value (Column O) calculated using inner bulge cell composition, the delta AIC (Column P) and p-value (Column Q) calculated using leukocyte cell composition, the delta AIC (Column R) and p-value (Column S) calculated using epidermis stem cell composition, the delta AIC (Column T) and p-value (Column U) calculated using merged epidermis basal cell composition, the q-values calculated using each cell composition (Columns V-AA), and the number of cell types with significant associations (Column AB). All the eGenes with only one significant cell type association were considered as “cell type-specific”, whereas all the eGenes with more than one significant cell type association were considered as “cell type-associated”.

## Supplementary Data 24 legend. eQTL results: GTEx skin at collapsed resolution

The ensemble gene ID (Column A), the gene name (Column B), the start (Column C) and end (Column D) of the gene, the gene ID (chromosome_start_end_genotype; Column E), variant position (Column F), reference allele (Column G), alternative allele (Column H), the reference SNP cluster ID (Column I), delta AIC (Column J) and p-value (Column K) calculated using collapsed epidermal cell composition, the delta AIC (Column L) and p-value (Column M) calculated using collapsed inner bulge cell composition, the delta AIC (Column N) and p-value (Column O) calculated using collapsed leukocyte composition, the q-values calculated using each cell composition (Columns P-R), and the number of cell types with significant associations (Column S). All the eGenes with only one significant cell type association were considered as “cell type-specific”, whereas all the eGenes with more than one significant cell type association were considered as “cell type-associated”.

## Supplementary Data 25 legend. Skin eQTL colocalization with skin GWAS traits results

For each of the 23 UK Biobank GWAS traits (Column A) that was colocalized with the eQTLs identified using six collapsed skin populations, we provide a description of the trait (Column B) and how we collapsed results from similar studies (Column C). This table further describes the gene ensemble IDs (Column D), the SNP ID (chromosome_start_end_genotype; Column E), and the number of SNPS tested for each gene (Column F). Results of the colocalization include the posterior probability of the model not sharing a signal (PP0; Column G), the posterior probability of only the eQTL having a signal (PP1; Column H), posterior probability of only the GWAS having a signal (PP2; Column I), posterior probability of both the GWAS and eQTL having a signal, but the causal variant is different (PP3; Column J), posterior probability of both the GWAS and eQTL having a shared causal variant (PP4; Column K), and the posterior probability of the SNP (Column L). We also include the p-value for each cell type used as covariates included in the model for the eQTL analysis: collapsed epidermal cells (Column M), inner bulge cell (Column N), and Leukocyte (Column O). Only eGenes with PP4 > 0.1 are shown.
