# Supplementary material for: Cellular deconvolution of GTEx tissues powers discovery of disease and cell-type associated regulatory variants
Source: Nat Commun. 2020 Feb 19;11:955. doi: 10.1038/s41467-020-14561-0 (PMC7031340; doi:10.1038/s41467-020-14561-0)
Supplement: Supplementary file 29 — Reporting Summary [file 41467_2020_14561_MOESM29_ESM.pdf]

nature research

Corresponding author(s): *Double-blind peer review submissions: write DBPR and your manuscript number here instead of author names.*

Last updated by author(s): *YYYY-MM-DD*

Reporting Summary

Nature Research wishes to improve the reproducibility of the work that we publish. This form provides structure for consistency and transparency in reporting. For further information on Nature Research policies, see Authors & Referees and the Editorial Policy Checklist .

Statistics

For all statistical analyses, confirm that the following items are present in the figure legend, table legend, main text, or Methods section.

n/a	Confirmed	
[ ]	[x]	The exact sample size (*n*) for each experimental group/condition, given as a discrete number and unit of measurement
[x]	[ ]	A statement on whether measurements were taken from distinct samples or whether the same sample was measured repeatedly
[ ]	[x]	The statistical test(s) used AND whether they are one- or two-sided<br>*Only common tests should be described solely by name; describe more complex techniques in the Methods section.*
[ ]	[x]	A description of all covariates tested
[ ]	[x]	A description of any assumptions or corrections, such as tests of normality and adjustment for multiple comparisons
[ ]	[x]	A full description of the statistical parameters including central tendency (e.g. means) or other basic estimates (e.g. regression coefficient) AND variation (e.g. standard deviation) or associated estimates of uncertainty (e.g. confidence intervals)
[ ]	[x]	For null hypothesis testing, the test statistic (e.g. *F*, *t*, *r*) with confidence intervals, effect sizes, degrees of freedom and *P* value noted<br>*Give P values as exact values whenever suitable.*
[x]	[ ]	For Bayesian analysis, information on the choice of priors and Markov chain Monte Carlo settings
[x]	[ ]	For hierarchical and complex designs, identification of the appropriate level for tests and full reporting of outcomes
[ ]	[x]	Estimates of effect sizes (e.g. Cohen's *d*, Pearson's *r*), indicating how they were calculated

Our web collection on statistics for biologists contains articles on many of the points above.

Software and code

Policy information about availability of computer code

Data collection	All data used in this study was obtained from publicly available resources. No software was used for data collection.
Data analysis	R version 3.5.1 (programming language); 10X Genomics Cell Ranger version 2.1.0 (set of analysis pipelines that process Chromium scRNA-seq output); Seurat version 3.0.2 (R package designed to analyze scRNA-seq data); CIBERSORT (R tool to estimate abundance of cell types in a mixed cell population); lme4 version 1.1 (R package for fitting linear and generalized linear mixed-effects models); pbkrtest version 0.4 (R tools to test mixed effect models); coloc version 3.1 (R tool to perform the colocalization of two genetic traits)

For manuscripts utilizing custom algorithms or software that are central to the research but not yet described in published literature, software must be made available to editors/reviewers. We strongly encourage code deposition in a community repository (e.g. GitHub). See the Nature Research guidelines for submitting code & software for further information.

Data

Policy information about availability of data

All manuscripts must include a data availability statement. This statement should provide the following information, where applicable:

- Accession codes, unique identifiers, or web links for publicly available datasets
- A list of figures that have associated raw data
- A description of any restrictions on data availability

Sequence data that support the findings of this study (all Figures) is available for human liver scRNA-seq (GSE11546); for human skin scRNA-seq (http://dom.pitt.edu/rheum/centers-institutes/scleroderma/systemicsclerosiscenter/database/); and for Tabula Muris mouse scRNA-seq (https://figshare.com/articles/Robject_files_for_tissues_processed_by_Seurat/5821263/1). Scripts to process, analyze, and generate Figures from the data is available at https://github.com/mkrdonovan/gtex_deconvolution. The source data underlying all Figures is available in the Source Data file (Tables 1-28).

Field-specific reporting

Please select the one below that is the best fit for your research. If you are not sure, read the appropriate sections before making your selection.

☒ Life sciences ☐ Behavioural & social sciences ☐ Ecological, evolutionary & environmental sciences

For a reference copy of the document with all sections, see nature.com/documents/nr-reporting-summary-flat.pdf

Life sciences study design

All studies must disclose on these points even when the disclosure is negative.

Sample size	We performed deconvolution on only the represented GTEx tissues represented by Tabula Muris.
Data exclusions	No data were excluded.
Replication	Confirmation of our ability to use signature genes based on expression profiles from mouse cell types for the deconvolution of the cellular composition of human GTEx tissues was performed using a first proof-of-concept tissue, liver, scRNA-seq and verified using a second proof-of-concept tissue, skin.
Randomization	No randomization was performed.
Blinding	Investigators were not blinded.

Reporting for specific materials, systems and methods

We require information from authors about some types of materials, experimental systems and methods used in many studies. Here, indicate whether each material, system or method listed is relevant to your study. If you are not sure if a list item applies to your research, read the appropriate section before selecting a response.

Materials & experimental systems

n/a	Involved in the study	
☒	☐	Antibodies
☒	☐	Eukaryotic cell lines
☒	☐	Palaeontology
☒	☐	Animals and other organisms
☒	☐	Human research participants
☒	☐	Clinical data

Methods

n/a	Involved in the study	
☒	☐	ChIP-seq
☒	☐	Flow cytometry
☒	☐	MRI-based neuroimaging
